# Oral bacterial composition associated with lung function and lung inflammation in a community-based Norwegian population

**DOI:** 10.1186/s12931-023-02491-6

**Published:** 2023-07-12

**Authors:** Rajesh Shigdel, Ane Johannessen, Huang Lin, Shyamal Peddada, Francisco Gómez Real, Tamar Ringel-Kulka, Cecilie Svanes, Randi Jacobsen Bertelsen

**Affiliations:** 1 Department of Clinical Science, University of Bergen, P.O. Box 7804, N-5020 Bergen, Norway; 2 Department of Global Public Health and Primary Care, University of Bergen, Bergen, Norway; 3 Eunice Kennedy Shriver National Institute of Child Health and Human Development, 6710B Rockledge Drive, Bethesda, MD 20892 USA; 4 Department of Gynecology and Obstetrics, Haukeland University Hospital, Bergen, Norway; 5 UNC Gillings School of Global Public Health, Department of Maternal and Child Health, The University of North Carolina at Chapel Hill, Chapel Hill, NC USA; 6 Department of Occupational Medicine, Haukeland University Hospital, Bergen, Norway; 7 Centre for International Health, Department of Global Public Health and Primary Care, University of Bergen, Bergen, Norway; 8 Oral Health Centre of Expertise in Western Norway, Bergen, Norway

**Keywords:** Oral microbiome, Lung function, Forced expiratory volume, Fractional exhaled nitric oxide, Forced vital capacity

## Abstract

**Background:**

The oral cavity is the gateway to the bacteria community in the lung. Disruption of the symbiotic balance of the oral microbiota has been associated with respiratory diseases. However, little is known about the relationship between oral bacteria and respiratory outcomes in the general population. We aimed to describe the associations between oral bacteria, lung function, and lung inflammation in a community-based population.

**Methods:**

Oral (gingival) samples were collected concurrently with spirometry tests in 477 adults (47% males, median age 28 years) from the RHINESSA study in Bergen, Norway. Bacterial DNA from the 16S rRNA gene from gingival fluid were sequenced by Illumina^®^MiSeq. Lung function was measured using spirometry and measurement of fractional exhaled nitric oxide (FeNO) were performed to examine airway inflammation. Differential abundance analysis was performed using ANCOM-BC, adjusting for weight, education, and smoking.

**Results:**

The abundance of the genera *Clostridiales, Achromobacter, Moraxella*, *Flavitalea* and *Helicobacter* were significantly different among those with low FEV_1_ (< lower limit of normal (LLN)) as compared to normal FEV_1_ i.e. ≥ LLN. Twenty-three genera differed in abundance between among those with low FVC < LLN as compared to normal FEV_1_ ≥ LLN. The abundance of 27 genera from phyla *Actinobacteria, Bacteroidetes, Firmicutes, Proteobacteria* and *Sacchribacteria* differed significantly between elevated FeNO levels (≥ 50 ppb) compared to FeNO ≤ 25 ppb.

**Conclusion:**

Oral bacterial composition was significantly different for those with low FEV or FVC as compared to those with normal lung function equal to or higher than LLN. Differential bacterial composition was also observed for elevated FeNO levels.

**Supplementary Information:**

The online version contains supplementary material available at 10.1186/s12931-023-02491-6.

## Introduction

Oral microbiome interacts with the gut and lung microbial communities and together they have an important role in human health and disease [1]. The relationship between the host and its microbiome is bidirectional and extremely dynamic [1], and has recently received considerable attention. The development and advancement of culture-independent techniques have revolutionized our understanding of the human microbiome and its role in health and disease by providing comprehensive and unbiased characterization of microbial community [2].

The oral cavity is the gateway for the microbiota of both the gut and the lungs [3]. Studies have shown that bacterial communities of healthy lung overlaps with those found in the mouth, but in lower concentrations [4]. In a healthy lung constant balance of the lung microbiota is maintained via microbial immigration and elimination [5, 6]. There is evidence showing that the oral microbiota is associated with periodontal diseases, cancer development, asthma, and chronic obstructive pulmonary disease (COPD), diabetes, obesity, and cardiovascular diseases [7, 8]. Furthermore, reduced microbial diversity and richness have been associated with changes in local cell functionality, immune response, and disease progression [9].

The oral microbiota may affect the microbial community in the lung through micro aspiration and mucosal dispersal [4]. Spirometry is an important tool in diagnosis and management of many pulmonary conditions. Spirometry is commonly interpreted in comparison with predicted normal values, based on a patient’s sex, height, age, and race, with the observed value expressed as percent of predicted [10]. Abnormal lung function have been defined as less than 80% of the predicted values, which helps to predict the risk and prognosis associated with lung disease [11]. Lee et al. [12] have reported that the microbial diversity positively correlated with lung function and the relative abundance of *Firmicutes* phyla; in particular the *Gemella* genus in sputum was negatively correlated with percent predicted FVC in young adults. In young subjects, the relative abundance of *Actinomyces* genus was inversely associated with absolute FEV_1_ but a similar association was not observed in older age, however the phylum *Actinobacteria* was positively associated with FVC. In cystic fibrosis patients with pulmonary exacerbation, a higher relative abundance of *Veillonella*, *Granulicatella* or *Prevotella* in sputum was associated with higher FEV_1_, while samples from patients with higher inflammation and higher relative abundance of *Pseudomonas* had lower FEV_1_ [13]. Several other studies have shown that bacterial genera *Streptococcus, Staphylococcus, Prevotella,* and *Gemella* in the lower airways are negatively associated with airways obstruction [14].

Most of the studies reporting associations between the upper respiratory tract microbiome and lung function have been conducted in patients with respiratory diseases. Emerging evidence indicates increasing relative abundance of *Proteobacteria* and *Bacteroidetes* phyla and lower abundance of *Moraxella* among asthmatic adults [15].

Measurement of fractional nitric oxide (FeNO) is a non-invasive procedure that may assess Type-2 airway inflammation, and that is sometimes used to assist in the diagnosis of asthma and to monitor treatment effects [16]. According to the American Thoracic Society (ATS) and European Respiratory Society (ERS) guidelines, exhalation flow of FeNO levels > 50 ppb is generally considered an indication of possible presence of Th2 inflammation [17]. One study reported an association between the indoor microbiome and FeNO levels [18], with increased bacterial alpha diversity and decreased fungal alpha diversity associated with higher FeNO. However, to the best of our knowledge no studies have so far investigated the association between oral microbiome with lung function and FeNO in a large population-based study. The purpose of the current study is to study the association of gingival bacterial diversity with lung function and FeNO level in a general population. In this current study we hypothesized that those with low lung function or those with high level of FeNO level will have differential abundance of gingival bacteria as compared to those with normal or higher lung function or those with low level of FeNO level.

## Materials and methods

The study population includes 477 adult participants (≥ 18 years of age) investigated as part of the RHINESSA generation study (www.rhinessa.net) in Bergen, Norway. The participants were examined in 2014–2015 with questionnaires, interviews, and clinical examinations. In this study pregnant women and participants who reported any respiratory infection two weeks before the clinical examination, or who had gone through any major surgery, or had comorbidities such as unstable angina, pneumonia were not included in the study. We collected extensive information, including information on age, gender, educational level, smoking status, and use of antibiotics in the four weeks before clinical examination. As antibiotics affect the composition of the microbiome, fourteen participants were excluded from further analyses due to the use of antibiotics in the four weeks before gingival sampling. The questionnaires are available at www.rhinessa.net.

### Lung function

Lung function measurements were assessed using a spirometer (EasyOne™ Spirometer) by trained health professionals, in line with the ATS and the ERS recommendations [19]. The maximum forced expiratory volume in one second (FEV_1_) and maximum forced vital capacity (FVC) of up to five technically acceptable maneuvers were selected, even if they did not come from the same maneuver, and the FEV_1_/FVC ratio was calculated from these. Measurements of height and weight were performed by the field workers prior to the lung function measurement. The lower limit of normal (LLN) values and percent predicted FEV_1_ (ppFEV_1_) and FVC (ppFVC) were obtained using the Global Lung function Initiative (GLI) spirometry reference equation [20]. FEV1, FVC < LLN was considered low lung function and greater than LLN was considered as normal. We performed sensitivity analysis further categorizing ppFEV1 and ppFVC into four groups, < 80%, 80–90%, 90–100% and ≥ 100% predicted values. Eighty percent predicted lung function is commonly used as the cut-off for detecting and classifying the severity of COPD, where 100% predicted reflects the average value expected in a healthy individual of any given size, age and sex [21].

### *Fractional exhaled nitric oxide*

Fractional exhaled nitric oxide (FeNO) measurements were performed according to standardized methods, using a electrochemical device (NIOX MINO, Aerocrine AB, Solna, Sweden) [22]. The first technically acceptable measurement was used for the analysis. FeNO is measured at the plateau of expiration and given in parts per billion (ppb). The participants with measured FeNO were categorized into three groups.

FeNO < 25 ppb, 25–50 ppb and FeNO > 50 ppb. The FeNO levels of less than 25 ppb were defined as normal levels and more than 50 ppb were defined as eosinophilic inflammation. We also performed sensitivity analysis using the FeNO levels of < 25 ppb defined as normal levels and ≥ 25 ppb was defined as high levels according to the ATS and European Respiratory Society guidelines [17].

### Gingival sample collection

The clinical examination included gingival fluid sampling on which 16S rRNA MiSeq amplicon sequencing was done on the Microbiome Core Facility, University of North Carolina, Chapel Hill, NC, USA. The description of the biosampling, the laboratory procedures and quality control are described in detail in the Additional file 1.

### Statistical analysis

Descriptive statistics are presented as mean ± standard deviation (SD) for continuous variables and as frequency (percentage) for categorical variables. Rarefaction of the data 184,528 sequences per sample was done to adjust for differences in library size across sample to aid comparisons of beta diversity. Alpha diversity at the genus level was calculated based on several indices (Shannon, Observed, Chao1 and Pileous evenness and coverage) and the alpha diversity differences between FVC, FEV_1_ low vs high, and FeNO categories were examined by Wilcoxon rank-sum test. The linear relationship between FVC and FEV_1_ and FeNO with alpha diversity was assessed using linear regression models.

Principal Coordinates Analysis (PCoA) was used to visualize beta diversity Bray Curtis distance matrices. Permutational multivariate analysis of variance (PERMANOVA) [23] and permutational analysis of multivariate dispersion (PERMDISP) [24], was applied to compare the distances among low FEV_1_, and FVC vs high FEV_1_, and FVC, and FeNO categories. The PERMDISP test gives information on whether the observed differences are due to different spatial medians or due to the heterogeneity of dispersions. The determination of differentially abundant bacterial genera between different categories of FVC, FEV_1_ and FeNO was performed using analysis of composition of microbiomes with bias correction (ANCOM-BC) [25] at genus level adjusting for age, height, weight, education, and smoking status. However, age, height, and gender are already accounted for in LLNFEV_1_ and LLNFVC calculation, so we did not adjust them in the percent predicted lung function models. All p-values were adjusted with the Benjamini-Hochberg (B-H) method to adjust for multiple comparisons and statistical significance was assessed with a threshold of False Discovery Rate (FDR) at 0.05.

We performed a pairwise comparison between lung function categories considering those with FEV_1_ and FVC low vs normal based on lower limit of normal. For the FeNO categories FeNO < 25 ppb was used as reference category. All statistical analyses were performed in R v.4.2.2, using the packages phyloseq (version 1.42.0)ggplot2 (version 3.4.2), microbiome version 1.20.0:(http://microbiome.github.io/microbiome/), vegan:(http://vegan.r-forge.r-project.org/), miaverse version1.6.0 https://microbiome.github.io/ and ANCOM-BC version 2.0.2 [25]. Beside ANCOM-BC we have performed two additional microbiome differential abundance methods, such as DESeq2 (version 3.17) and LEfSe (version 1.10.0).

## Results

### Study population characteristics

A total of 477 adults aged between 18 and 47 years of age from the RHINESSA study in Bergen, Norway, were included in the analyses (Table 1). The women (n = 223) were slightly younger than the men (n = 254) (mean age: 27 and 29 years, respectively). Women had higher educational level, lower BMI and were less likely to be current smokers as compared to the male participants p < 0.05 (Table 1). The mean (SD) FVC in women was 3.39 L (± 0.52) and 5.57 L (± 0.76) in men and the mean (SD) FEV_1_ in women and men were 3.25 L (± 0.43) and 4.49 L (± 0.65), respectively. The mean (SD) FeNO levels were higher in men (24 ppb (± 15) than in women (18 ppb (± 13)) (Table 1).

**Table 1:** Demographics and lung functions of study total population and gender comparisons (p-value for test for difference between men and women, t-test for continuous variables and Chi-square test for categorical variables)

	All (n = 477)	Women (n = 223)	Men (n = 254)	p-value
Age, (year)				0.018
Range	18, 47	18, 45	18, 47	
Mean (SD)	28 (7)	27 (7)	29 (7)	
BMI, (kg/m^2^),				< 0.001
Range	17.1, 46.5	17.2, 42.9	17.1, 46.5	
Mean (SD)	25.1 (4.6)	24.0 (4.5)	26.1 (4.4)	
Batch, n (%)				0.800
Old	279 (58)	132 (59)	147(58)	
New	198 (42)	91(41)	107 (42)	
Smoking, n (%)				0.30
Never	334 (70)	163 (73%)	171 (67%)	
Previous	77 (16)	30 (13%)	47 (19%)	
Current	66 (14)	30 (13%)	36 (14%)	
Education				0.009
Primary	10 (2)	2 (0.9%)	8 (3.4%)	
Secondary	179 (40)	74 (35%)	105 (45%)	
Tertiary	259 (58)	138 (64%)	121 (52%)	
FVC (L)				< 0.001
Range	1.52, 8.28	1.52, 5.74	3.8, 8.28	
Mean (SD)	4.81(1.05)	3.93 (0.52)	5.57 (0.76)	
FVC				
Low	7 (2.8%)	10 (4.5%)	17 (3.6%)	
Normal	246 (97.2%)	211 (94.6%)	457 (96.0%)	
FEV1(L)				< 0.001
Range	1.18, 6.79	1.18–4.55	2.70–6.79	
Mean (SD)	3.91 (0.83)	3.25(0.43)	4.49(0.65)	
FEV1				
Low	16 (6.3%)	15 (6.7%)	31 (6.5%)	
Normal	237 (93.7%)	206 (92.4%)	443 (93.1%)	
FeNO (ppb)				< 0.001
Range	5, 158	5, 123	5, 158	
Mean (SD)	21 (14)	18 (13)	24 (15)	
FeNO categories				< 0.001
< 25 ppb	345 (73%)	186(83.4%)	159 (62.8%)	
25–49 ppb	113 (24%)	30 (13.5%)	83 (32.8%)	
$\ge$ 50 ppb	15 (3%)	4 (1.8%)	11 (4.3%)	

Values are n (%) or mean ± SD

*FVC* forced vital capacity, *FEV*_*1*_ forced expiratory volume in 1 s, *ppFVC* Percentage predicted forced vital capacity, *ppFEV*_*1*_ percentage predicted forced expiratory volume, *FeNO* fractional exhaled nitric oxide

### Gingival bacterial community profiling

**Fig. 1:**
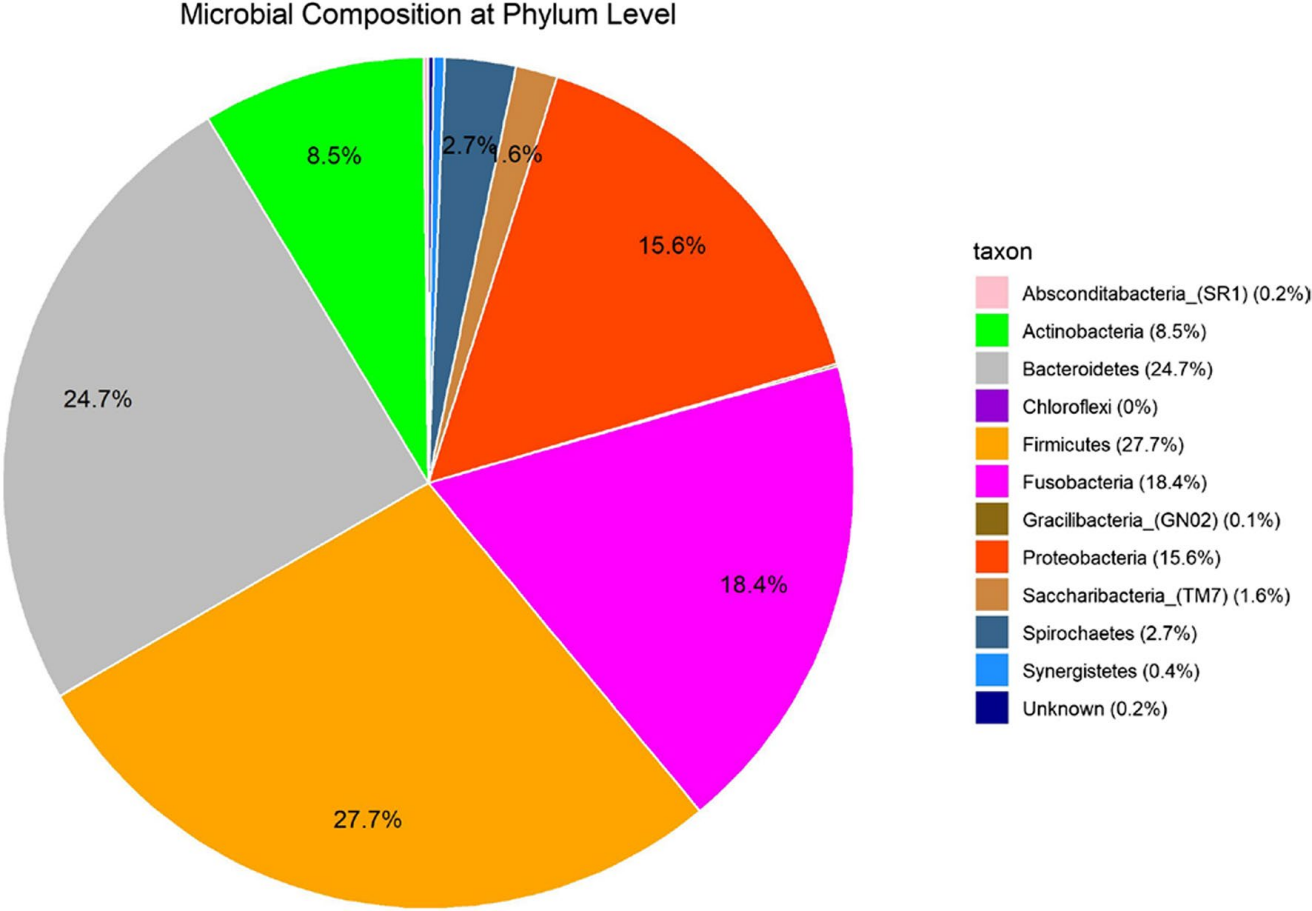
Microbial composition by phylum level

For all samples combined the most prevalent bacterial phyla contributing to the gingival fluid samples were *Firmicutes* (27.8%), *Bacteriodetes* (24.8%), *Fusobacteria* (18.5%), *Proteobacteria* (15.6%) and *Actinobacteria* (8.5%) (Fig. 1). For genera level *Fusobacterium* (15.2%, phylum: *Fusobacteria), Streptococcus (9.7%, phylum: Firmicutes),* and *Prevotella* (8.4%, phylum: *Bacteroidetes)* were the most prevalent bacteria and were present in all the gingival samples Fig. 1.

### Bacterial diversity, spirometry and FeNO

**Fig. 2:**
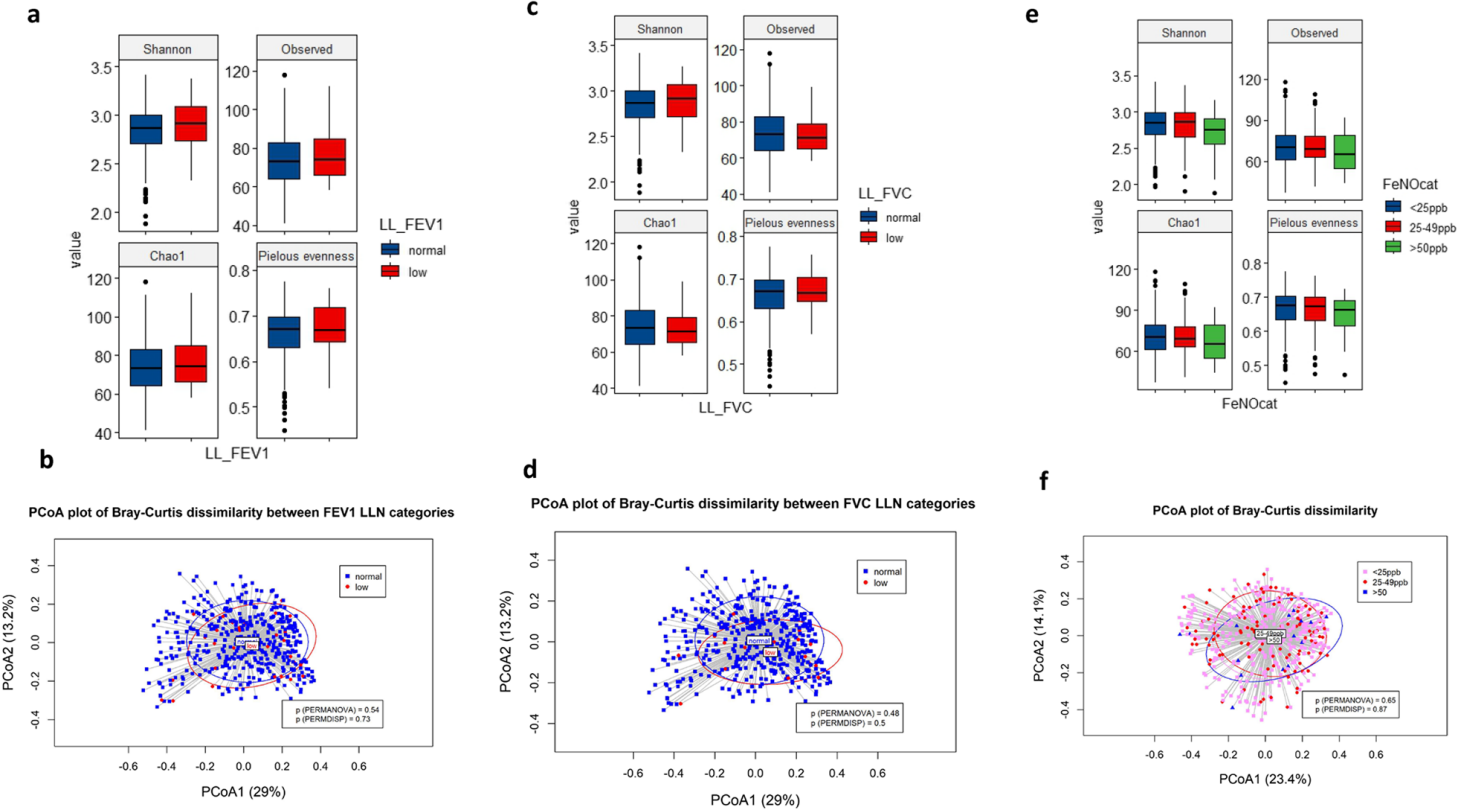
**a, c, e** Alpha diversity comparison between different FVC, FEV_1_, and FeNO categories using Kruskal Wallis test Boxplots represent the median and interquartile range (IQR) with whiskers extending to the minimum/maximum value, but no longer than 1.5 × IQR. **b, d** Principal coordinate analysis (PCoA) plot based on the bray–curtis distance matrix constructed using ASVs. The percentage of variability explained by the corresponding coordinate is indicated on the axes. Each point represents a single sample—blue symbols indicate sample with FVC or FVC ≥ LLN, red symbols indicate samples with FVC or FEV1, < LLN, The lines indicate vectors representing the relationships between ASVs and each sample category. The ellipses serve a visual guide to group differences. Comparison of beta diversity between different categories showed no significant differences in community structure (p > 05). **f** Principal coordinate analysis (PCoA) plot based on the bray–curtis distance matrix constructed using ASVs. The percentage of variability explained by the corresponding coordinate is indicated on the axes. Each point represents a single sample– pink symbols indicate sample with FeNO < 25 ppb, red symbols indicate samples with FeNO level between 25–49 ppb, and and blue symbols those with FeNO level > 50 ppb. The lines indicate vectors representing the relationships between ASVs and each sample category. The ellipses serve a visual guide to group differences. Comparison of beta diversity between different categories showed no significant differences in community structure (p > 05)

There was no significant difference in alpha diversity (Kruskal Wallis test) between FEV_1_, FVC and FeNO categories (Fig. 2a, c, e). In linear regression model the alpha diversity index, Chao1, was positively associated with both FEV_1_ and FVC (p < 0.05). (Table 2); whereas FEV_1_, and FVC was negatively associated with the Pileous evenness (p < 0.05) (Table 2). Shannon and chao1 diversity index decreased with increasing FeNO levels in women (p < 0.05), but not for men (Fig. 2c). For beta-diversity (between group comparison), there was no statistically significant difference in beta diversity between the FVC, FEV_1_ and FeNO groups (Fig. 2a, b, f).

**Table 2:** Linear regression between alpha diversity values, lung function (FEV_1_ and FVC) and FeNO

Variables	Shannon index			Chao			Pielous evenness		
Total	Effect estimate	SE	p-value	Effect estimate	SE	p-value	Effect estimate	SE	p-value
FVC	− 0.016	0.011	0.188	1.82	0.799	0.023	− 0.01	0.002	0.005
FEV_1_	− 0.016	0.015	0.261	2.12	1.011	0.036	− 0.01	0.001	0.015
FeNO	− 0.042	0.024	0.081	− 1.48	1.640	0.368	− 0.01	0.004	0.083

*FVC* forced vital capacity, *FEV*_*1*_ forced expiratory volume in 1 s, *FeNO* fractional exhaled nitric oxide

### *Comparison of oral bacterial communities by FVC and FEV*_*1*_* categories*

**Fig. 3:**
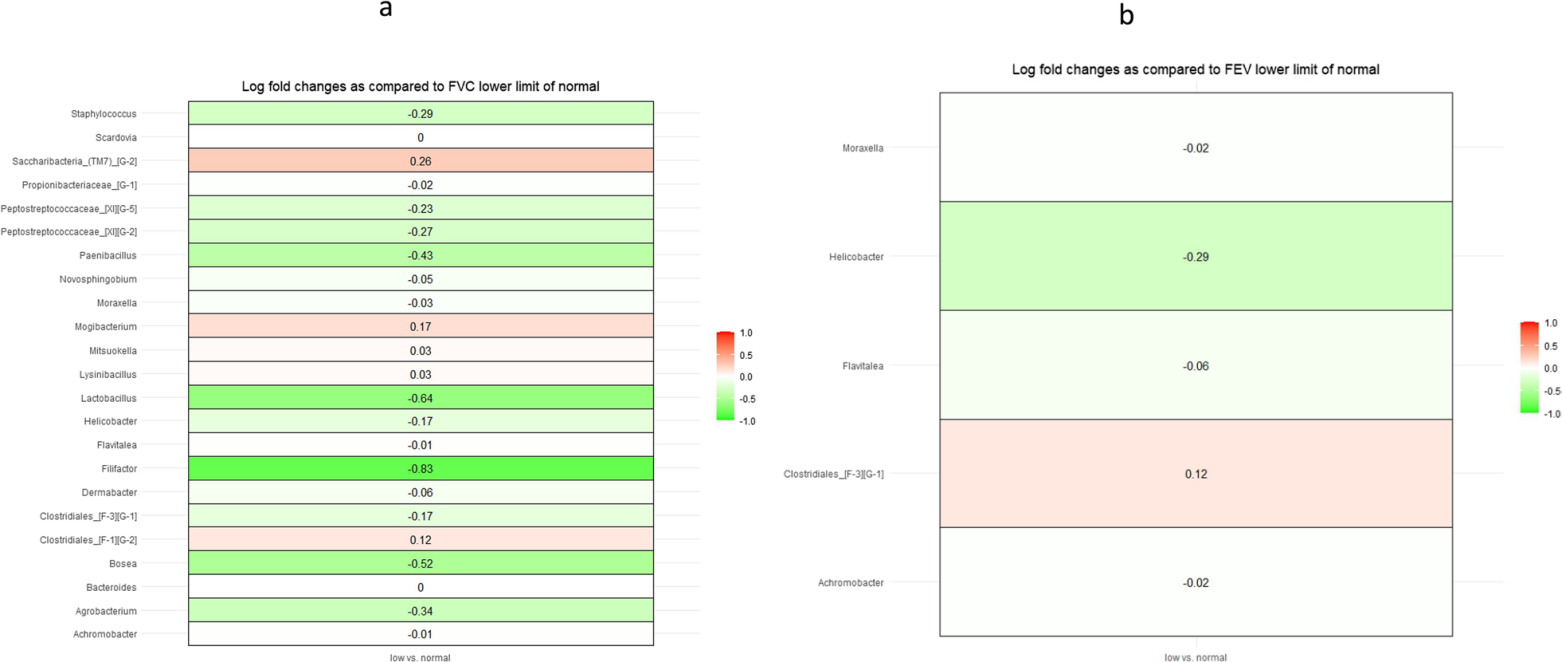
**a** Figure showing overview of gingival bacteria genera that differ in abundance between those with FVC defined as ‘normal’ (FVC ≥ LLN) according to height, age, sex and ethnicity (reference group) compared to those with lower FVC (< LLN FVC). **b** Figure showing overview of gingival bacteria genera that differ in abundance between those with FEV_1_ defined as ‘normal’ (≥ LLN) according to the values expected for their height, age, sex, and ethnicity (reference group) compared to those with lower FEV_1_ (< LLN FEV_1_)

ANCOM-BC was used to assess the differentially abundant bacteria taxa (at genus level) between the Low FVC (< LLN, FEV_1_(< LLN) groups vs those with normal lung function (≥ LLN). Five genera, *Clostridiales [F-3], Achromobacter, Moraxella* and *Helicobacter and Flavitalea* differed in abundance in the low FEV1 as compared to those with normal FEV1. (p < 0.001) (Fig. 3b). As compared to those with normal FEV1, the lower FEV_1_ (< LLN) groups had higher abundance of the genera *Clostridiales [F-3].* (Table 3). There were 23 bacteria genera that differed significantly in abundance between those with normal FVC as compared to those with normal FVC (Fig. 3a). Five genera Acrombacter, *Clostridiales [F-3], mroxella, Helicobaacter and Flavitalea were differentially abundance among those* with lower FEV1 or FVC as compared to normal FEV1 or FVC.

**Table 3:** Absolute abundance in gingival bacteria (genus) between participants with normal or high lung function (≥ LLN%, reference group) FVC and FEV_1_ vs those with low lung function (< LLN%)

Genus	Phylum	Sample with bacteria present %	FVC < LLN (n = 17)
			LFC
Staphylococcus	Firmicutes	21	− 0.29
Scardovia	Actinobacteria	19	0.001
Saccharibacteria_(TM7)_[G-2]	*Saccharibacteria*	19	0.26
Propionibacteriaceae_[G-1]	Actinomycetia	17	− 0.02
Peptostreptococcaceae_[XI][G-2]	*Firmicutes*	24	− 0.23
Peptostreptococcaceae_[XI][G-5]	*Firmicutes*	14	− 0.27
Paenibacillus	Firmicutes	17	− 0.43
Novosphingobium	*Saccharibacteria*	12	− 0.05
Moraxella	*Proteobacteria*	11	− 0.03
Mogibacterium	*Firmicutes*	12	0.17
Mitsuokella	Firmicutes	16	0.03
Lysinibacillus	*Firmicutes*	11	0.03
Lactobacillus	*Firmicutes*	10	− 0.64
Helicobacter	Proteobacteria	12	− 0.17
Flavitalea	Bacteroidetes	9	− 0.01
Filifactor	Firmicutes	36	− 0.83
Dermabacter	Actinobacteria	11	− 0.06
Clostridiales_[F-3][G-1]	Firmicutes	9	0.17
Clostridiales_[F-1][G-2]	Firmicutes	12	− 0.12
Bosea	Proteobacteria	24	− 0.52
Bacteroides	Bacteroidetes	13	0.001
Agrobacterium	*Saccharibacteria*	25	− 0.34
Achromobacter	Proteobacteria	10	− 0.01

Genus	Phylum	%	FEV_1_ < LLN (n = 31)
			LFC
*Clostridiales [F-3][G-1]*	*Firmicutes*	9	0.12
*Achromobacter*	*Proteobacteria*	10	− 0.02
*Moraxella*	*Proteobacteria*	11	− 0.02
*Helicobacter*	*Proteobacteria*	12	− 0.29
*Flavitalea*	*Bacteroidetes*	10	− 0.02

ANCOM-BC test, adjusted for weight, smoking and education

*ppFVC* Percentage predicted forced vital capacity, *ppFEV*_*1*_ percentage predicted forced expiratory volume, *LFC* log fold change, *SE* standard error

In a sensitivity analysis we found the same bacteria that differed significantlyn abundance (low FVC vs normal) among those with ppFVC < 80% as compared with those with ppFVC ≥ 100%, similarly, we found same bacteria that significantly differed in abundance (low FVC vs normal) among those with ppFEV1 < 80% as compared with those with ppFEV1 ≥ 100%.

In addition to ANCOMBC, we have performed a sensitivity an analysis using two other differential abundance methods Lefse and DESEq2 and found one bacteria genus *Desulfobulbus* to be significantly lower in the group with low FEV_1_ and FVC, whereas genus *Abiotrophia* was significantly different among those with low FEV_1_ vs Normal FEV_1._ Lefse did not detect any statistically significant difference between FVC groups.

### Comparison of oral bacterial communities by FeNO categories

**Fig. 4:**
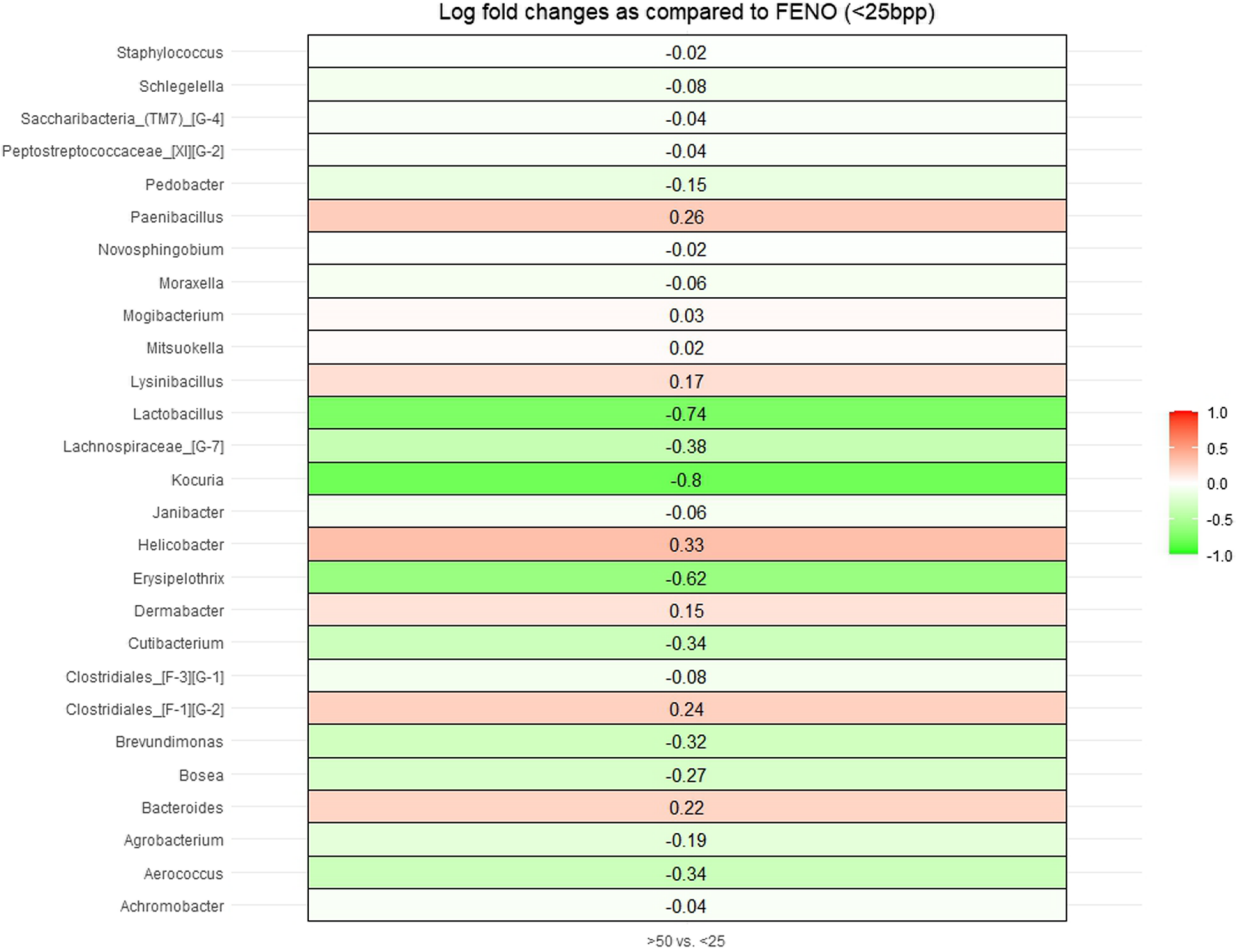
Figure showing overview of gingival bacteria genera that differ in abundance between those with low FeNO levels (≤ 25 ppb) (reference group) compared to those with high FeNO levels (> 50 ppb)

According to ANCOM-BC 27 bacteria genera differed significantly in abundance between the high FeNO category (> 50 ppb) and the low FeNO group (≤ 25 ppb) (Fig. 4). Most of these bacteria belong to the phyla *Firmicutes, Proteobacteria, Bacteroides, Actinobacteria* and *Saccharibacteria* (Table 4). In a sensitivity analysis, we did not find a statistically significant difference in the abundance of bacteria low FeNO group (≤ 25 ppb) as compared to those with higher FeNO level (> 25 ppb).

**Table 4:** Difference in absolute abundance of bacteria genera between participants with low FeNO level (< 25 ppb, reference group) vs high FeNO group

Genera	Phylum	Sample with bacteria present %	FeNO > 50 ppb (n = 15)	
			LFC	SE
*Kocuria*	*Actinobacteria*	37	− 0.80	0.28
*Dermabacter*	*Actinobacteria*	11	0.15	0.26
*Janibacter*	*Actinobacteria*	23	− 0.07	0.35
*Cutibacterium*	*Actinobacteria*	36	− 0.34	0.29
*Bacteroides*	*Bacteroidetes*	13	0.22	0.33
*Pedobacter*	*Bacteroidetes*	24	− 0.15	0.26
*Lysinibacillus*	*Firmicutes*	11	0.17	0.26
*Paenibacillus*	*Firmicutes*	17	0.26	0.42
*Staphylococcus*	*Firmicutes*	21	− 0.02	0.25
*Aerococcus*	*Firmicutes*	26	− 0.34	0.21
*Lactobacillus*	*Firmicutes*	10	− 0.74	0.31
*Clostridiales_[F-1][G-2]*	*Firmicutes*	12	0.24	0.35
*Clostridiales_[F-3][G-1]*	*Firmicutes*	9	− 0.08	0.19
*Lachnospiraceae_[G-7]*	*Firmicutes*	25	− 0.38	0.26
*Mogibacterium*	*Firmicutes*	12	0.03	0.20
*Peptostreptococcaceae_[XI][G-2]*	*Firmicutes*	24	− 0.04	0.23
*Erysipelothrix*	*Firmicutes*	38	− 0.62	0.29
*Mitsuokella*	*Firmicutes*	16	0.02	0.30
*Brevundimonas*	*Proteobacteria*	24	− 0.32	0.31
*Bosea*	*Proteobacteria*	24	− 0.27	0.33
*Agrobacterium*	*Proteobacteria*	25	− 0.19	0.35
*Novosphingobium*	*Proteobacteria*	12	− 0.02	0.22
*Achromobacter*	*Proteobacteria*	10	− 0.04	0.20
*Schlegelella*	*Proteobacteria*	22	− 0.08	0.23
*Helicobacter*	*Proteobacteria*	12	0.33	0.29
*Moraxella*	*Proteobacteria*	11	− 0.06	0.20
*Saccharibacteria_(TM7)[G-4]*	*Saccharibacteria*	29	− 0.04	0.43

ANCOM-BC test, adjusted for age, gender, smoking, and BMI

*FeNO* fractional exhaled nitric oxide, *LFC* log fold change, *SE* Standard error

## Discussion

In this study we explored the composition of oral bacteria as related to lung function in a community-based general adult population. The same five bacteria genera were differentially abundance among those with either low FEV1 or FVC. Genera acrombacter*,* moraxella*, helicobacter and flavitalea those belongs to phylum proteobacteria were significantly lower abundance among those* with lower FEV1 or FVC as compared to normal FEV1 or FVC, whereas genera *Clostridiales_[F-3][G-1],* phylum Firmicutes was significantly higher among those with lower FEV1 or FVC. The types of bacteria identified by differential abundance analysis differed significantly between persons with low lung function as compared to optimal lung function (≥ LLN). Most bacteria in the phyla *Actinobacteria, Firmicutes, Bacteroidetes,* and *Proteobacteria* were of significantly lower abundance in the group with the lowest lung function. We did not observe significant differences in beta diversity across the different lung function categories, but the bacterial diversity decreased with increasing FeNO level, in women only, and 27 bacteria genera differed in abundance between the low and high FeNO categories. These bacteria belong to the *Actinobacteria, Bacteroidetes, Firmicutes, Proteobacteria,* and *Saccharibacteria* phyla. To the best of our knowledge, this is the first study investigating the association of oral microbiome with lung function and FeNO in the general population.

The abundance of the bacteria genera *Moraxella*, and *Achromobacter* was lower in those from the low lung function (FVC for *Moraxella* and FEV1 for *Achromobacter*) and the high FeNO group. This may be an indication that these oral bacteria may play a role in lung inflammation and may therefore explain the link between oral bacteria composition and low lung function. Furthermore, the abundance of *Moraxella* was higher in those with low FEV_1._ Moraxella is a gram-negative coccus, aerobic and initially thought to be a harmless commensal bacterium of the upper respiratory tract but lately recognized as an important for upper respiratory tract infection in children [26]. *Achromobacter* is a gram-negative bacteria known to colonize the respiratory tract of cystic fibrosis patients; these bacteria are intrinsically resistant to several antibiotics [27].

We do not have a thorough dental examination and thus cannot exclude the possibility of periodontal disease such as gingivitis and periodontitis being present. These conditions are characterized by inflamed gums caused by inflammatory bacteria. These could potentially reach the lower respiratory tract through micro aspiration and systemic dissemination and could cause lung inflammation and thus explain the association with low lung function. However, as reported in our previous paper, few of the study participants scored high on the Community Periodontal Index, which is a marker of periodontal health status [28]. The association between periodontal health status and lung function has been previously described for this cohort and it was found that poor periodontal health was associated with increasing airways obstruction [28].

In our previous paper where we have looked at the association of oral hygiene habit with self-reported gingival bleeding we found that self-reported gingival bleeding was associated with a higher abundance of well-known and novel periodontal pathogens such as Porphyromonas endodontalis, Treponema denticola, and Fretibacterium spp., these bacteria are members of the Red complex [29]. However, it was observed that the abundance of bacteria belonging to the gram-positive phyla Firmicutes and Actinobacteria was lower in these cases. On the other hand, individuals who engaged in flossing and rinsed with mouthwash twice daily displayed a higher overall abundance of bacteria in the Proteobacteria phylum. Unfortunately, we do not have information on the use of immune modulatory medications so we could not adjust or stratify our analysis based on use of immune modulatory medication. In this study population only 4.6% (n = 22) participants were taking asthma medication and in general we did not see any difference in lung function between those without or those using asthma medication. This is most likely because it is a general population sample without any cases of severe asthma. We did not observe any difference in oral bacteria by use of asthma medication during the last 12 months.

In the present study *Bacteroides* spp. was present in higher abundance in the high FeNO category. Given the inflammatory potential of the bacteria within the *Bacteroides* genus, this fits well with its presence in those with high FeNO levels, as this is a marker for lung inflammation.

The abundance of the genera *Lysinibacillus*, *Mogibacterium,* and *Clostridiales[F-1][G-1]* was higher in those with high FeNO level. Higher prevalence of *Lysinibacillus* have been reported to be associated with endodontic infection [30] and both *Mogibacteria* and *Lysinibacillus* can lead to caries progression [31]. *Clostridiales* allow other bacteria to grow, and they are known periodontal pathogens [32]. *Dermabacter* was high in those with high levels of FeNO. *Dermabacter* is a gram-positive rod, considered a human skin colonizer, however, in immunocompromised patients with severe comorbidities *Dermabacter hominis* is considered as a relevant pathogen [33].

Oral microbiota plays an important role in the natural history of many respiratory diseases. Oral and upper airways have direct communication with the lungs and the movement of commensals or the bacteria that reside in the oral cavity into the lungs, has been reported in multiple studies [34–36]. There are several suggested mechanisms for how oral pathogens can affect lung health, such as the concept of “The Oral Lung Axis” where researchers have proposed oral health status as a determinant of lung health [36]. With dysbiosis in the oral cavity, dental plaque increases together with the colonization of oral opportunistic pathogens such as gram-negative bacilli. Some of the latter are also known respiratory pathogens [37]. The opportunistic pathogens present in the periodontal pocket, such as in particular anaerobic bacteria, can enter the lower airway through the process of micro aspiration. In the case of oral microbial dysbiosis, translocation of bacteria and bacterial metabolites could modulate the host immune response through dendritic cells in lungs leading to lung inflammation. Vicious cycle hypothesis suggests that to clear pathogenic bacteria, intermittent signaling occurs when bacteria interact via pathogen recognition receptors on airway epithelial and immune cells that leads to chronic inflammation in the lung [38]. The association between respiratory disease and oral health has also already been shown by systematic reviews and meta-analyses [35, 39]. Several possible mechanisms have been proposed to explain the potential role of oral bacteria in the pathogenesis of respiratory infection, such as of oral pathogens, role of periodontal disease-associated enzymes, and role of cytokines originating from periodontal tissues that promote infections by respiratory pathogens by altering lung epithelial cells [40]. Therefore, we can consider oral health status as a potential method to assess and maybe even predict respiratory health status, including lung function. Longitudinal studies may be warranted for further investigation of this connection. Understanding the microbiota characteristics and its relationship with lung function can potentially improve respiratory health by targeting oral bacteria identified to induce inflammation in the lungs and offer great promise to improve the health of people living with impaired lung function.

The strength of this study is the well-defined large population study with extensive data from questionnaires and interviews as well as multiple clinical outcomes, including lung function and FeNO, and biological samples. A potential weakness of this study was that the gingival samples were analysed in two different batches (different time points for microbiome sequencing). However, the ANCOM-BC method is robust for batch effect, and it was also adjusted in the other statistical models. Though not incorporated in the current study, measures of inflammation markers and immune response could certainly help to better understand the role of oral microbiome in relation to lung function.

## Conclusion

Studying a community-based young adult population, our results suggest that the composition of the oral microbiome differ across the different levels of lung function and lung inflammation as reflected in FeNO. Further studies with metagenomic approaches are needed to understand the functional activity and pathophysiological mechanism of the microbiome in order to explain the underlying nature of the association between the oral microbiome and lung function. Longitudinal studies may shed light on the timing and progression of such changes and better help understand the patho-mechanism and causality of these changes.

## Supplementary Information

**Additional file 1.** Summplementary methods.

## Data Availability

The datasets presented in this study can be found in online repositories. The Dryad repository, accessible at: 10.5061/dryad.r2280gbfh.
