# Supplementary material for: Oral bacterial composition associated with lung function and lung inflammation in a community-based Norwegian population
Source: Respir Res. 2023 Jul 12;24:183. doi: 10.1186/s12931-023-02491-6 (PMC10337198; doi:10.1186/s12931-023-02491-6)
Supplement: Supplementary file 1 — Additional file 1. Summplementary methods. [file 12931_2023_2491_MOESM1_ESM.docx]

**Gingival fluid sampling**

The time of day for collection of gingival fluid samples was recorded, together with information of whether the participants were fasting before sample collection. Gingival fluid was collected with sterile paper points (PROTAPER, Jacobsen Dental) from the gingival crevice (area between the gingiva crest and the neck of the tooth) at five per-protocol predetermined sites in the lower and upper jaw; between the two frontal teeth, left frontal tooth (lateral side), right frontal tooth (lateral side), left molar number 6 (facing molar 5), and right molar number 6, facing molar number 5. Sterile procedures were applied with sterile mirror and tweezers, gloves, and surgical face mask. The paper points were frozen directly (-80°C) after collection in 2 ml Microtubes safelock Biopur tubes without buffer; with five paper points (from upper or the lower jaw) stored in one tube.

*16S rRNA gene amplicon sequencing*

Bacterial DNA was extracted from the gingival fluid samples and based on all five paper points from the lower jaw. Overall, 12.5 ng of total DNA was amplified using a combination (4:1) of Universal and Bifidobacterium-specific primers targeting the V1-V2 region of the bacterial 16S rRNA gene (Edwards, Rogall et al. 1989, Fierer, Hamady et al. 2008). Primer sequences contained overhang adapters appended to the 5’ end of each primer for compatibility with Illumina sequencing platform. The complete sequences of the primers were:

8F - 5’ TCGTCGGCAGCGTCAGATGTGTATAAGAGACAGAGAGTTTGATCCTGGCTCAG3’

BifidoF-5’ TCGTCGGCAGCGTCAGATGTGTATAAGAGACAGAGGGTTCGATTCTGGCTCAG3’

338R - 5’ GTCTCGTGGGCTCGGAGATGTGTATAAGAGACAGGCTGCCTCCCGTAGGAGT3’.

Master mixes contained 12.5 ng of total DNA, 0.2 µM of each primer and 2x KAPA HiFi HotStart ReadyMix (KAPA Biosystems, **Wilmington, MA).** The thermal profile for the amplification of each sample had an initial denaturing step at 95°C for 3 minutes, followed by a cycling of denaturing of 95°C for 30 seconds, annealing at 55°C for 30 seconds and a 30 second extension at 72°C (25 cycles), a 5 minute extension at 72°C and a final hold at 4°C. Each 16S amplicon was purified using the AMPure XP reagent (Beckman Coulter, Indianapolis, IN). In the next step each sample was amplified using a limited cycle PCR program, adding Illumina sequencing adapters and dual‐index barcodes (index 1(i7) and index 2(i5)) **(Illumina, San Diego, CA)** to the amplicon target. The thermal profile for the amplification of each sample had an initial denaturing step at 95°C for 3 minutes, followed by a denaturing cycle of 95°C for 30 seconds, annealing at 55°C for 30 seconds and a 30 second extension at 72°C (8 cycles), a 5 minute extension at 72°C and a final hold at 4°C. The final libraries were again purified using the AMPure XP reagent (Beckman Coulter), quantified and normalized prior to pooling. The DNA library pool was then denatured with NaOH, diluted with hybridization buffer and heat denatured before loading on the MiSeq reagent cartridge **(Illumina) and on the MiSeq instrument (Illumina). Automated cluster generation and paired–end sequencing with dual reads were performed** according to the manufacturer’s instructions.

*Sequencing data analysis*

Multiplexed paired-end fastq files were produced from the sequencing results of the Illumina MiSeq using the Illumina software configure BclToFastq. The paired-end fastqs were joined into a single multiplexed, single-end fastq using the software tool fastq-join. Demultiplexing and quality filtering was performed on the joined results. Quality analysis reports were produced using the FastQC software. Bioinformatics analysis of bacterial 16S amplicon sequencing data was conducted using the Quantitative Insight Into Microbial Ecology (QIIME) software version 2017.3.0(1). Amplicon sequence variant (ASV) was performed on the quality filtered results.  Chimeric sequences were detected and removed using ChimeraSlayer. To assign taxonomy, we used the Human Oral Microbiome Database (www.homd.org), which is a library that contains genomes from the human oral cavity (2). Total of 47443921 reads were obtained for 477 subjects by v1-v2 16S RNA sequencing. An average number of 99463 (from 10017 to 292207) reads per samples was used for downstream analysis. 242 distinct bacterial ASVs were identified.

1. Caporaso JG, Kuczynski J, Stombaugh J, Bittinger K, Bushman FD, Costello EK, et al. QIIME allows analysis of high-throughput community sequencing data. Nature methods. 2010;7(5):335-6.

2. Chen T, Yu WH, Izard J, Baranova OV, Lakshmanan A, Dewhirst FE. The Human Oral Microbiome Database: a web accessible resource for investigating oral microbe taxonomic and genomic information. Database : the journal of biological databases and curation. 2010;2010:baq013.
